# Expression of the transient receptor potential channels TRPV1, TRPA1 and TRPM8 in mouse trigeminal primary afferent neurons innervating the dura

**DOI:** 10.1186/1744-8069-8-66

**Published:** 2012-09-12

**Authors:** Dongyue Huang, Shuyang Li, Ajay Dhaka, Gina M Story, Yu-Qing Cao

**Affiliations:** 1 Washington University Pain Center and Department of Anesthesiology, Washington University School of Medicine, St. Louis, MO 63110, USA; 2 Department of Biological Structure, University of Washington, Seattle, WA, 98195, USA

**Keywords:** Headache, Migraine, Trigeminal ganglion, Dural afferent neuron, Transient receptor potential channel

## Abstract

**Background:**

Migraine and other headache disorders affect a large percentage of the population and cause debilitating pain. Activation and sensitization of the trigeminal primary afferent neurons innervating the dura and cerebral vessels is a crucial step in the “headache circuit”. Many dural afferent neurons respond to algesic and inflammatory agents. Given the clear role of the transient receptor potential (TRP) family of channels in both sensing chemical stimulants and mediating inflammatory pain, we investigated the expression of TRP channels in dural afferent neurons.

**Methods:**

We used two fluorescent tracers to retrogradely label dural afferent neurons in adult mice and quantified the abundance of peptidergic and non-peptidergic neuron populations using calcitonin gene-related peptide immunoreactivity (CGRP-ir) and isolectin B4 (IB4) binding as markers, respectively. Using immunohistochemistry, we compared the expression of TRPV1 and TRPA1 channels in dural afferent neurons with the expression in total trigeminal ganglion (TG) neurons. To examine the distribution of TRPM8 channels, we labeled dural afferent neurons in mice expressing farnesylated enhanced green fluorescent protein (EGFPf) from a TRPM8 locus. We used nearest-neighbor measurement to predict the spatial association between dural afferent neurons and neurons expressing TRPA1 or TRPM8 channels in the TG.

**Results and conclusions:**

We report that the size of dural afferent neurons is significantly larger than that of total TG neurons and facial skin afferents. Approximately 40% of dural afferent neurons exhibit IB4 binding. Surprisingly, the percentage of dural afferent neurons containing CGRP-ir is significantly lower than those of total TG neurons and facial skin afferents. Both TRPV1 and TRPA1 channels are expressed in dural afferent neurons. Furthermore, nearest-neighbor measurement indicates that TRPA1-expressing neurons are clustered around a subset of dural afferent neurons. Interestingly, TRPM8-expressing neurons are virtually absent in the dural afferent population, nor do these neurons cluster around dural afferent neurons. Taken together, our results suggest that TRPV1 and TRPA1 but not TRPM8 channels likely contribute to the excitation of dural afferent neurons and the subsequent activation of the headache circuit. These results provide an anatomical basis for understanding further the functional significance of TRP channels in headache pathophysiology.

## Background

Migraine and other primary headache disorders affect a large proportion of the general population and often cause debilitating pain. A crucial step in the pathogenesis of a headache attack is the activation and sensitization of primary afferent neurons (PANs) in the trigeminovascular system [1-3]. These neurons are pseudounipolar cells, with somata localized in the trigeminal ganglion (TG) and giving rise to one fiber from which both the central and peripheral projections derive. The peripheral fibers innervate the dura mater and cerebral blood vessels, and the central fibers project to the upper cervical and medullary dorsal horn. Nociceptive signals originate from the activation of various chemo- and mechano-sensors at the peripheral terminals of PANs. Subsequently, the afferent activity reaches the central terminals of PANs and activates second-order neurons in the cervical/medullary dorsal horn, from which the signals are conveyed to the thalamus and eventually reach the cortex, where the perception of headache is formed. Understanding the expression pattern of chemo-sensing molecules in the PANs of the headache circuit will add to our understanding of headache pathophysiology and has the potential to facilitate the development of new therapeutics.

Transient receptor potential (TRP) channels are a large family of non-selective cation channels. Several TRP channel family members, including TRP cation channel subfamily V member 1 (TRPV1), subfamily A member 1 (TRPA1) and TRP channel melastatin 8 (TRPM8), are expressed in distinct populations of PANs and are activated in response to both temperature changes and a broad spectrum of endogenous and exogenous chemical ligands [4]. Numerous functional studies have suggested that these TRP channels mediate hyperalgesia following tissue and nerve injury and therefore may represent potential targets for novel analgesic drugs [5]. Thus, it is important to investigate the contribution of these TRP channels to the activation of PANs in the headache circuit.

In rats, nerve fibers in the dura mater exhibit TRPV1-immunoreactivity (TRPV1-ir) [6]. In addition, 97% of dural afferent fibers in the guinea pig respond to capsaicin [7]. However, the effects of TRPV1 antagonists have been inconsistent in various *in vivo* models of headache [8-10]. Mustard oil, a TRPA1 agonist, evoked inward currents in 42% of dural afferent neurons in rats [11]. TRPA1 activation has also been shown to mediate dural vasodilation induced by exposure to nasal irritants [12,13]. It is unclear whether nasal irritants activate TRPA1 channels in the dural afferent neurons. In fact, the expression of TRPA1 channels in dural afferent neurons has not been investigated. One hypothesis is that nasal irritants excite dural afferent neurons via intraganglionic neurotransmission [14-17]. The irritants would first activate TRPA1 channels on PANs innervating the nasal mucosa, leading to spike generation. Subsequently, this afferent activity would result in the release of neurotransmitters and neuropeptides from the somata of nasal afferent neurons [18-22]. This, in turn, cross-excite nearby dural afferent neurons within the TG. However, the spatial distribution of TRPA1-expressing (TRPA1^+^) neurons in the TG has not been studied, nor do we know their spatial association with dural afferent neurons. Likewise, whether TRPM8 channels are expressed in dural afferent neurons and, if so, whether they play a role in the activation of the trigeminovascular system has not been investigated. It is also important to characterize both the spatial distribution of TRPM8-expressing neurons in the TG and their relationship with dural afferent neurons.

Various genetically modified mouse models offer great tools to study the functional significance of TRP channels in headache pathophysiology. Nevertheless, the majority of studies regarding the subpopulations of TG neurons that project to the dura and cerebral vessels were conducted in rats and cats. Given the well-documented differences between rats and mice with respect to the expression of two commonly used PAN population markers, calcitonin gene-related peptide (CGRP) and isolectin B4 (IB4) [23], it is important to quantitatively assess the abundance of TG neuron subpopulations in dural afferents to gain insight into headache mechanisms using mouse models.

In the present study, we used two fluorescent tracers to retrogradely label dural afferent neurons in adult mice. We quantified the abundance of peptidergic and non-peptidergic populations within dural afferents using CGRP-immunoreactivity (CGRP-ir) and IB4 binding, respectively. We also compared the expression patterns of TRPV1, TRPA1 and TRPM8 channels in dural afferent neurons with their patterns in the total TG neuron population. Our results show that a substantial fraction of dural afferent neurons bind IB4. Surprisingly, the percentage of dural afferent neurons that exhibit somatic CGRP-ir is only half that the percentage of the total TG neuron population. We also found that both TRPV1 and TRPA1 channels are expressed in dural afferent neurons. Using nearest-neighbor measurement, we predicted that TRPA1^+^ TG neurons are clustered around a subset of dural afferent neurons and therefore may have a higher probability of cross-excitation within the TG. Interestingly, TRPM8-expressing TG neurons are virtually absent in the dural afferent population, nor do they cluster around dural afferent neurons in TG. This lack of small-diameter TRPM8-expressing neurons may partially account for the larger sizes of dural afferent neurons relative to those of the total TG population.

## Results

### Localization and size distribution of dural afferent neurons in the TG

**Figure 1:**
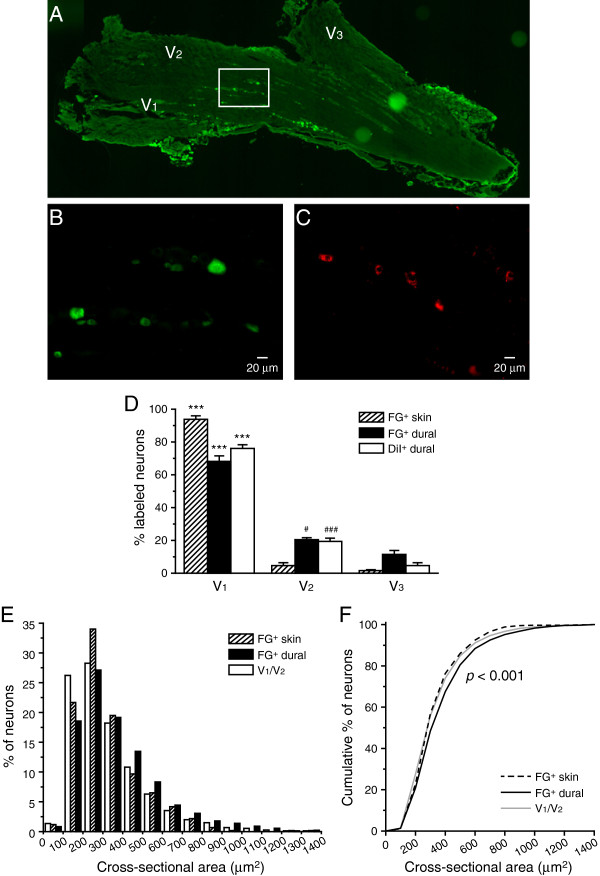
**Localization and size distribution of the TG neurons innervating the dura and periorbital skin.** (**A**) Representative image of a TG section showing FG-labeled (FG^+^) dural afferent neurons. Note that the labeled neurons are distributed predominantly within the V_1_ division and to some extent in the V_2_ division. (**B**) High-magnification image of the region indicated in (**A**). (**C**) Representative image of DiI-labeled (DiI^+^) dural afferent neurons. (**D**) The distributions of FG^+^ and DiI^+^ in the skin and dural afferent neurons from the three TG divisions (n = 3–4 mice in each group; on average, 200 labeled neurons from each mouse were counted). The majority of labeled neurons are distributed in the V_1_ and V_2_ divisions (one-way ANOVA with post hoc Bonferroni test, *** *p* < 0.001, V_1_ versus V_2_ or V_3_ distribution in each group; ^#^*p* < 0.05, ^###^*p* < 0.001, V_2_ versus V_3_ distribution in each group). (**E**) Histogram of the size distributions of total TG neurons in the V_1_/V_2_ divisions, FG^+^ skin afferent neurons, and FG^+^ dural afferent neurons (n = 2316, 600 and 2208 neurons pooled from three mice, respectively). (**F**) Cumulative distributions of the cross-sectional areas of total TG neurons in the V_1_/V_2_ divisions, FG^+^ skin afferent neurons, and FG^+^ dural afferent neurons (the same neurons as in **E**). The sizes of dural afferent neurons are significantly larger than those of the skin afferents and the V_1_/V_2_ TG neurons (*p* < 0.001, Kruskal-Wallis ANOVA with Dunn’s post hoc test).

To label dural afferent neurons, we applied the fluorescent tracer Fluorogold (FG) to the dura above a section of the superior sagittal sinus (SSS) in adult mice [24]. Retrogradely labeled neurons were observed in the bilateral TG. First, we examined the distribution of labeled neurons in the ophthalmic (V_1_), maxillary (V_2_) and mandibular (V_3_) divisions of the TG. Consistent with previous reports [25,26], we found that the majority (~70%) of FG-labeled neurons were localized in the V_1_ division of the TG, whereas only a small percentage of labeled neurons were distributed in the V_2_ and V_3_ divisions (Figure 1A, B and D, black bars, *p* < 0.001, one-way ANOVA with post hoc Bonferroni test). To confirm this result, we retrogradely labeled dural afferent neurons with another fluorescent tracer, 1,1'-dioctadecyl-3,3,3',3'-tetramethylindocarbocyanine perchlorate (DiI) [27]. The distribution of DiI-labeled dural afferents was similar to the distribution of FG-labeled neurons, with more than 70% of the labeled neurons localized in the V_1_ division of the TG (Figure 1C and D, open bars, *p* < 0.001, one-way ANOVA with post hoc Bonferroni test, V_1_ versus V_2_ or V_3_ distribution in each group).

Next, we compared the size distribution of dural afferent neurons with the distribution of neurons in the V_1_/V_2_ divisions of the TG. The mean cross-sectional area of the V_1_/V_2_ neurons was 327 ± 4 μm^2^ (n = 2208 neurons pooled from three mice). In contrast, the mean cross-sectional area of the FG-labeled dural afferents was 374 ± 5 μm^2^ (n = 2316 neurons pooled from three mice), which was significantly larger than that of the V_1_/V_2_ TG neurons (Figure 1E, F, *p* < 0.001, Mann–Whitney *U* test). This result is in agreement with previous reports regarding the size distribution of PANs innervating the dura and intracranial vasculature in rats [25,28,29]. 

It is possible that FG may preferentially label the TG neurons that have a larger soma diameter, thereby skewing our size comparison between dural afferent neurons and V_1_/V_2_ TG neurons. To address this possibility, we labeled TG neurons innervating the periorbital skin (between the eyes) with an intradermal injection of FG. As with the dural afferents, more than 90% of the FG-labeled skin afferent neurons were localized in the V_1_ division of the TG (Figure 1D, hatched bars, *p* < 0.001, one-way ANOVA with post hoc Bonferroni test). The mean cross-sectional area of the skin afferent neurons was 319 ± 25 μm^2^ (n = 600 neurons pooled from three mice), which is similar to the V_1_/V_2_ TG neurons (*p* = 0.8), but was significantly smaller than the FG-labeled dural afferents (Figure 1E, F, *p* < 0.001, dural versus skin afferents or versus V_1_/V_2_ TG neurons, Kruskal-Wallis ANOVA with Dunn’s post hoc test). We therefore conclude that FG labels TG neurons of various soma sizes with comparable efficiency.

**Figure 2:**
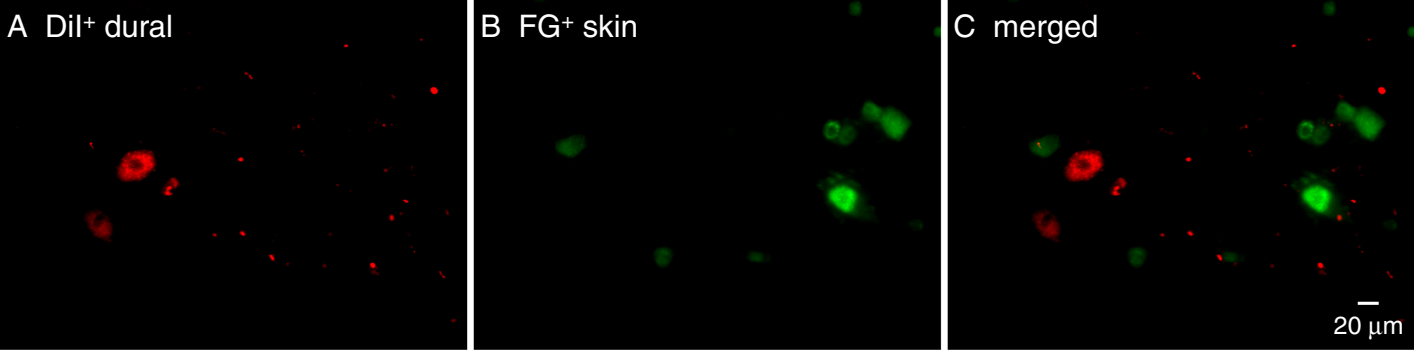
**Retrograde labeling of dural and facial skin afferent neurons from the same mouse.** Representative images of a TG section showing DiI^+^ dural afferent neurons (**A**) and FG^+^ neurons innervating the periorbital skin (**B**). Note that there is no overlap between the dural and skin afferent neurons as seen in the merged image (**C**) A total of 425 dural and 360 skin afferent neurons from three mice were counted, respectively).

Some recent studies have shown that individual TG neurons contain collaterals that project to both the meninges and extracranial tissue such as the skull and muscle [30,31]. We applied DiI to the dura above the SSS and injected FG into the periorbital skin to label both dural and skin afferent neurons in individual mice. We found no overlap between DiI-labeled dural afferent neurons (n = 425 neurons pooled from three mice) and FG-labeled skin afferents (n = 360 neurons pooled from three mice, Figure 2). Our result is consistent with a previous study showing little overlap between the TG neurons that innervate the middle cerebral artery and the forehead skin in adult rats [25]. 

### The distribution of dural afferent neurons expressing CGRP

**Figure 3:**
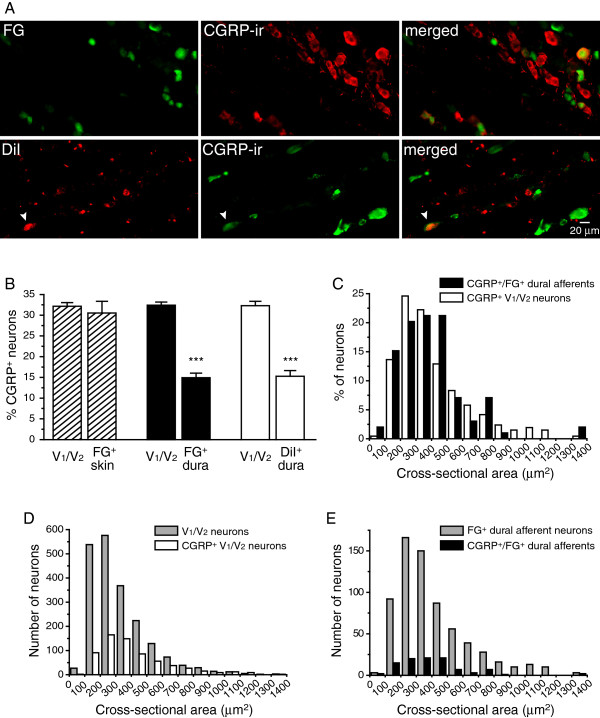
**The distribution of dural afferent neurons expressing the neuropeptide CGRP. **(**A**) Representative images of TG sections containing FG^+^ or DiI^+^ dural afferent neurons and neurons exhibiting CGRP-ir. Arrowheads indicate double-labeled FG^+^/CGRP^+^ and DiI^+^/CGRP^+^ dural afferent neurons. (**B**) The abundances of CGRP^+^ neurons in the V_1_/V_2_ divisions of the TG, in the FG^+^ skin afferent neurons, and in the FG^+^ or DiI^+^ dural afferent neurons (n = 3 mice in each group; *** *p* < 0.001, two-tailed *t*-test). On average, 685 V_1_/V_2_ neurons from each mouse were counted in each group. On average, 296 FG^+^ skin afferent neurons, 233 FG^+^ dural afferents, and 285 DiI^+^ dural afferent neurons were counted from each mouse. (**C**) The size distribution of the CGRP^+^ dural afferent neurons (filled bars, n = 99 neurons pooled from three mice) is similar to that of the CGRP^+^ neurons in the V_1_/V_2_ divisions of the TG (open bars, n = 661 neurons pooled from three mice, *p* = 0.7, Mann–Whitney *U* test). (**D**) The size distribution of the CGRP^+^ neurons (open bars, the same neurons as in **C**) and total neurons in the V_1_/V_2_ divisions of the TG (grey bars, n = 2041 neurons pooled from three mice). (**E**) The size distribution of the CGRP^+^ dural afferent neurons (filled bars, the same neurons as in **C**) and total FG^+^ dural afferents (grey bars, n = 700 neurons pooled from three mice).

We went on to examine the abundance of TG neurons subpopulations in the dural afferents. The neuropeptide CGRP plays an important role in migraine pathophysiology [32,33], and previous studies have shown that the meninges and cerebral arteries in rodents are densely innervated by CGRP-expressing (CGRP^+^) TG neurons [29,34-36]. The population of TG neurons that project to the cerebral vasculature contains a higher percentage of CGRP^+^ neurons compared with the entire TG [37]. Accordingly, we labeled dural afferent neurons with FG and stained TG sections using an anti-CGRP antibody (Figure 3A). CGRP-ir was observed in 32.4 ± 0.8% of the neurons in the V_1_/V_2_ divisions of the TG (Figure 3B, middle plot). Surprisingly, only 14.9 ± 1.1% of FG-labeled dural afferent neurons displayed CGRP-ir, which was significantly lower than that of V_1_/V_2_ neurons (*p* < 0.001, Figure 3B, middle plot). 

Our results are in apparent disagreement with a previous study of the enrichment of CGRP^+^ neurons in TG population projecting to the cerebral vasculature [37]. One possibility may be that relative to other TG neurons, CGRP^+^ TG neurons may be less efficient at taking up FG at their terminals and/or transporting FG to the soma. We labeled TG neurons innervating the periorbital skin with FG and stained the TG sections using the CGRP antibody. The percentage of CGRP^+^ neurons in the FG-labeled skin afferents (30.5 ± 2.8%) was comparable to that of V_1_/V_2_ neurons (32.2 ± 0.9%, Figure 3B, left plot), indicating that FG labels CGRP^+^ TG neurons as effectively as those that do not express CGRP.

To test whether CGRP^+^ dural afferent neurons take up and/or transport FG less efficiently than other fluorescent tracers, we retrogradely labeled dural afferent neurons with DiI and stained TG tissues using the CGRP antibody. To better preserve the DiI signal, the concentration of the detergent Triton X-100 in the solutions was decreased from 0.3% to 0.03% [38]. CGRP-ir was observed in 32.3 ± 1.1% of the V_1_/V_2_ TG neurons (Figure 3B, right plot), indicating that the low triton concentration did not compromise the sensitivity of the immunostaining. Only 15.3 ± 1.4% DiI-labeled dural afferent neurons exhibited CGRP-ir (*p* < 0.001, Figure 3B, right plot), which is in agreement with the results obtained from the FG-labeled dural afferent neurons. Taken together, we conclude that the percentage of dural afferent neurons exhibiting somatic CGRP-ir is significantly smaller than that of neurons in the V_1_/V_2_ TG divisions.

Both small- (< 600 μm^2^ cross-sectional area) and medium-sized (600–1400 μm^2^ cross-sectional area) TG neurons expressed CGRP (Figure 3C, D, open bars). The mean cross-sectional area of the CGRP^+^ neurons in the V_1_/V_2_ TG divisions was 417 ± 14 μm^2^ (n = 661 neurons pooled from three mice). The mean cross-sectional area of the CGRP^+^ neurons innervating the dura was 373 ± 10 μm^2^ (n = 99 neurons pooled from three mice; Figure 3C, E, black bars), which was comparable to the size of the CGRP^+^ neurons in the V_1_/V_2_ TG divisions (*p* = 0.7, Mann–Whitney *U* test, Figure 3C). Therefore, both small- and medium-sized dural afferent neurons express CGRP, albeit all at a lower abundance relative to the total TG neuronal population.

### The distribution of dural afferent neurons that bind IB4

**Figure 4:**
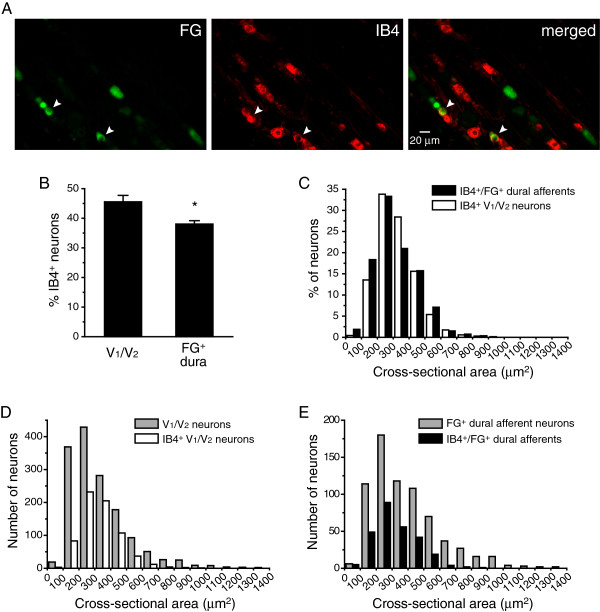
**The distribution of IB4**^**+**^**neurons in the V**_**1**_**/V**_**2**_**TG divisions and the dural afferent population. **(**A**) Representative images of a TG section containing FG^+^ dural afferent neurons and IB4-labeled neurons. Arrowheads indicate neurons that are both FG^+^ and IB4^+^. (**B**) The percentages of V_1_/V_2_ TG neurons and FG^+^ dural afferent neurons that are IB4^+^ (n = 3 mice; on average, 500 V_1_/V_2_ neurons and 235 FG^+^ neurons were counted from each mouse; * *p* < 0.05, two-tailed *t*-test). (**C**) The size distributions of the IB4^+^/FG^+^ dural afferent neurons (black bars, n = 267 neurons pooled from three mice) are similar to the distributions of the IB4^+^ neurons in the V_1_/V_2_ divisions of the TG (white bars, n = 686 neurons pooled from three mice, *p* = 0.1, Mann–Whitney *U* test). (**D**) The sizes of IB4^+^ neurons (open bars, the same neurons as in **C**) are significantly smaller than those of the V_1_/V_2_ neurons (grey bars, n = 1499 neurons pooled from three mice, *p* < 0.001, Mann–Whitney *U* test). (**E**) The sizes of IB4^+^/FG^+^ dural afferents (black bars, the same neurons as in **C**) are significantly smaller than those of the total FG^+^ dural afferent neurons (grey bars, n = 704 neurons pooled from three mice, *p* < 0.001, Mann–Whitney *U* test).

IB4 binding is commonly used to define the non-peptidergic population of primary afferents, i.e., sensory neurons that express little or very low levels of neuropeptides [39,40]. We labeled dural afferent neurons with FG and stained the sections with Alexa Fluor 594-conjugated IB4 (Figure 4A). In the V_1_/V_2_ divisions of the TG, 45.5 ± 2.2% of the neurons were labeled with IB4 (IB4^+^). The percentage of IB4^+^ neurons in the dural afferents was 38.0 ± 0.7%, significantly lower than that in the V_1_/V_2_ divisions (Figure 4B, *p* < 0.05). The size distribution of the IB4^+^ dural afferents was similar to the total IB4^+^ population of neurons in the V_1_/V_2_ divisions of the TG (*p* = 0.1, Mann–Whitney *U* test, Figure 4C). 

The mean cross-sectional area of the IB4^+^ neurons in the V_1_/V_2_ divisions of the TG was 327 ± 14 μm^2^ (n = 686 neurons pooled from three mice). More than 95% of the IB4^+^ neurons had a cross-sectional area smaller than 600 μm^2^, which is consistent with previous studies and indicated that these neurons belong to the small-sized TG population (Figure 4D, *p* < 0.001 compared with total V_1_/V_2_ TG neurons, Mann–Whitney *U* test). The mean cross-sectional area of the IB4^+^ neurons in the dural afferents was 316 ± 13 μm^2^ (n = 267 neurons pooled from three mice), which was significantly smaller than that of the entire FG-labeled population (Figure 4E, *p* < 0.001, Mann–Whitney *U* test).

### The distribution of TRPV1 channels in TG and dural afferent neurons

TRPV1 channels can be activated by noxious heat as well as by chemical ligands, including capsaicin, anandamide, and protons [41]. TRPV1-ir and sensitivity to capsaicin have been reported in dural afferent neurons from rats and guinea pigs, respectively [6,7]. Here, we investigated whether the distribution of TRPV1 channels in dural afferent neurons differs from their distribution in total TG tissue in mice.

**Figure 5:**
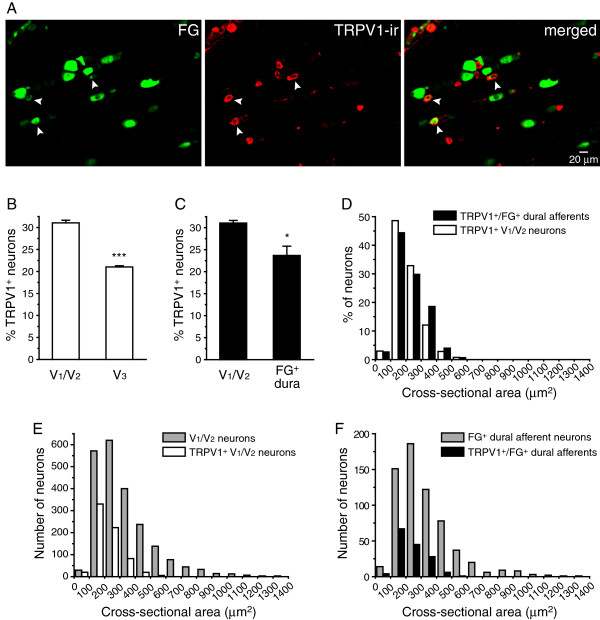
**The distribution of neurons expressing TRPV1 channels in the TG and dural afferent neuron populations. **(**A**) Representative images of a TG section containing FG^+^ dural afferent neurons and neurons exhibiting TRPV1-ir. Arrowheads indicate neurons that are both FG^+^ and TRPV1^+^. (**B**) The percentage of TRPV1^+^ TG neurons in the V_1_/V_2_ and V_3_ divisions (n = 3 mice; on average, 730 V_1_/V_2_ neurons and 500 V_3_ neurons were counted from each mouse; *** *p* < 0.001, two-tailed *t*-test). (**C**) The percentages of V_1_/V_2_ TG neurons (the same data set as in **B**) and FG^+^ dural afferent neurons that are TRPV1^+^ (n = 3 mice, on average, 213 FG^+^ neurons were counted from each mouse; * *p* < 0.05, two-tailed *t*-test). (**D**) The size distribution of TRPV1^+^/FG^+^ dural afferent neurons (n = 151 neurons pooled from three mice) are similar to the TRPV1^+^ neurons in the V_1_/V_2_ divisions of the TG (n = 679 neurons pooled from three mice, *p* = 0.1, Mann–Whitney *U* test). (**E**) The sizes of TRPV1^+^ neurons (the same neurons as in **D**) are significantly smaller than those of the neurons in the V_1_/V_2_ divisions of the TG (n = 2189 neurons pooled from three mice, *p* < 0.001, Mann–Whitney *U* test). (**F**) The sizes of TRPV1^+^/FG^+^ dural afferents (the same neurons as in **D**) are significantly smaller than those of the total FG^+^ dural afferent neurons (n = 638 neurons pooled from three mice, *p* < 0.001, Mann–Whitney *U* test).

We labeled the dural afferent neurons with FG and stained TG sections with an antibody against TRPV1 channels (Figure 5A). TRPV1-ir was present in 31.0 ± 0.6% of the neurons in the V_1_/V_2_ divisions of the TG, a significantly higher percentage than in the V_3_ division (21.0 ± 0.3%, *p* < 0.001, Figure 5B). In addition, 23.7 ± 2.1% of the FG-labeled dural afferent neurons exhibited TRPV1-ir (Figure 5C), in line with a previous study [6]. The percentage of TRPV1-expressing (TRPV1^+^) neurons in the dural afferent neurons was significantly lower than in the total V_1_/V_2_ TG population (Figure 5C, *p* < 0.05). 

The size distribution of the TRPV1^+^ dural afferents was similar to that the distribution of the TRPV1^+^ neurons in the V_1_/V_2_ divisions of the TG (*p* = 0.1, Mann–Whitney *U* test, Figure 5D). The mean cross-sectional area of the TRPV1^+^ neurons in V_1_/V_2_ was 215 ± 4 μm^2^ (n = 679 neurons pooled from three mice), which was significantly smaller than that of the total V_1_/V_2_ TG neuron population (327 ± 11 μm^2^, n = 2189 neurons pooled from three mice, *p* < 0.001, Mann–Whitney *U* test, Figure 5E). This finding is consistent with previous reports showing that TRPV1 is expressed almost exclusively in small-diameter c-fiber neurons [42,43]. The mean cross-sectional area of the TRPV1^+^ dural afferent neurons (228 ± 5 μm^2^, n = 151 neurons pooled from three mice) was also significantly smaller relative to the entire FG-labeled population (325 ± 13 μm^2^, n = 638 neurons pooled from three mice, Figure 5F, *p* < 0.001, Mann–Whitney *U* test).

### The distribution of TRPA1 channels in TG neurons and dural afferent neurons

TRPA1, another TRP channel family member, has been reported to sense noxious cold stimuli [44,45] (but see [46]). In addition, previous studies have shown that TRPA1 channels act as the sensor of a broad spectrum of endogenous compounds as well as environmental irritants [47-50]. The TRPA1 agonist mustard oil evokes inward currents in a subset of rat dural afferent neurons [11], and recent studies have shown that the intranasal application of TRPA1 agonists induces dural vasodilation [12,13]. Here, we quantified the distribution of TRPA1 channels in mouse dural afferent neurons.

**Figure 6:**
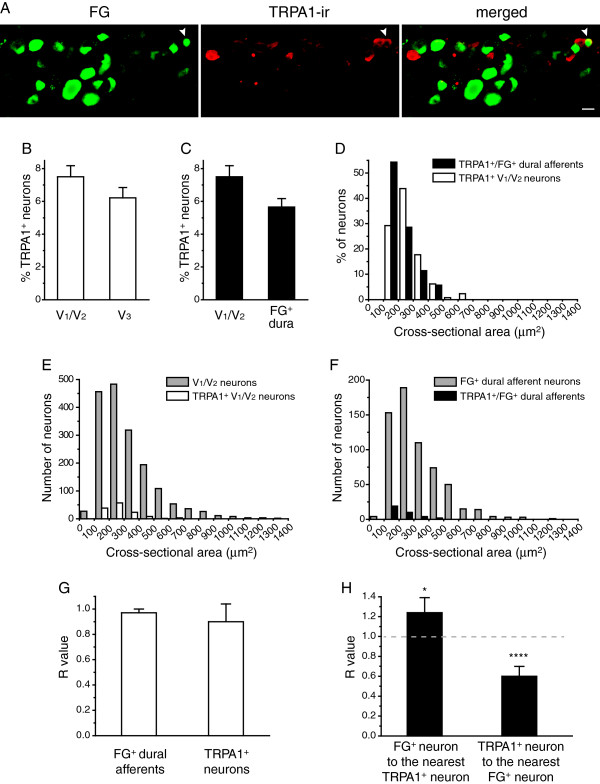
**The distribution of neurons expressing TRPA1 channels in the TG and dural afferent neurons. **(**A**) Representative images of a TG section containing a FG^+^/TRPA1^+^ dural afferent neuron (indicated by the arrowheads; scale bar: 20 μm). (**B**) The percentage of TRPA1^+^ TG neurons in the V_1_/V_2_ and V_3_ divisions (n = 3 mice; on average, 576 V_1_/V_2_ neurons and 428 V_3_ neurons were counted from each mouse). (**C**) The percentages of TRPA1^+^ V_1_/V_2_ TG neurons (the same data as in **B**) and FG^+^ dural afferent neurons (n = 3 mice, on average, 206 FG^+^ neurons were counted from each mouse). (**D**) The size distribution of TRPA1^+^/FG^+^ dural afferent neurons are significantly smaller than those of the TRPA1^+^ neurons in the V_1_/V_2_ divisions of the TG (n = 35 and 130 neurons pooled from three mice, respectively; *p* < 0.05, Mann–Whitney *U* test). (**E**) The sizes of TRPA1^+^ neurons (the same neurons as in **D**) are significantly smaller than those of the neurons in the V_1_/V_2_ divisions of the TG (n = 1729 neurons pooled from three mice, *p* < 0.001, Mann–Whitney *U* test). (**F**) The sizes of TRPA1^+^/FG^+^ dural afferents (the same neurons as in **D**) are significantly smaller than those of the total FG^+^ dural afferent neurons (n = 620 neurons pooled from three mice, *p* < 0.001, Mann–Whitney *U* test). (**G**) Nearest-neighbor measurement shows that both FG^+^ dural afferent neurons and TRPA1^+^ neurons are randomly distributed in the TG (n = 130 TRPA1^+^ neurons and 620 FG^+^ dural afferent neurons from 3 mice, the same cells as in **D** and **F**). **(H)** A modified nearest-neighbor measurement shows that TRPA1^+^ neurons are clustered around some, but not all, of the FG^+^ dural afferent neurons (the same neurons as in **G**).

We labeled the dural afferent neurons with FG and stained TG sections with anti-TRPA1 antibodies (Figure 6A, [51]). Within the three TG divisions, TRPA1-immunoreactivity (TRPA1-ir) was distributed uniformly in a very small population of TG neurons (Figure 6B). We found that 5.7 ± 0.5% of the FG-labeled dural afferent neurons were positive for TRPA1-ir, which is similar to the percentage of TRPA1^+^ neurons in the total V_1_/V_2_ TG neuron population (7.5 ± 0.7%, Figure 6C). 

The mean cross-sectional area of the TRPA1^+^ neurons in V_1_/V_2_ was 262 ± 8 μm^2^ (n = 130 neurons pooled from three mice), which is significantly smaller than that of the total V_1_/V_2_ TG neurons (325 ± 11 μm^2^, n = 1729 neurons pooled from three mice, *p* < 0.001, Mann–Whitney *U* test, Figure 6E). In fact, 97% of the TRPA1^+^ neurons had a cross-sectional areas that was smaller than 500 μm^2^ (Figure 6D, E, open bars), which is consistent with previous reports indicating that TRPA1 is predominantly expressed in small-diameter primary afferent neurons [43,45-47,51,52] (but see [53]). The mean cross-sectional area of the TRPA1^+^ dural afferent neurons (222 ± 9 μm^2^, n = 35 neurons pooled from three mice) was significantly smaller than that of the entire FG-labeled neuron population (324 ± 20 μm^2^, n = 620 neurons pooled from three mice, Figure 6F, *p* < 0.001, Mann–Whitney *U* test). Interestingly, the sizes of the TRPA1^+^ dural afferent neurons were also significantly smaller than those of the TRPA1^+^ neurons in the V_1_/V_2_ divisions of the TG (Figure 6D, *p* < 0.05, Mann–Whitney *U* test).

Recent studies have shown that the intranasal administration of TRPA1 agonists stimulates CGRP release and increases meningeal blood flow, suggesting that these events may contribute to the onset of headaches triggered by environmental irritants [12,13]. It has been suggested that TRPA1^+^ neurons innervating the nasal mucosa may cross-excite nearby dural afferent neurons within the TG via intraganglionic neurotransmission [14-22]. Here, we used nearest-neighbor measurement to determine whether TRPA1^+^ neurons are clustered around dural afferent neurons (or vice versa) [54,55].

First, we tested whether dural afferent neurons are randomly distributed in the V_1_/V_2_ divisions of the TG or are clustered. Thus, for each FG-labeled neuron, we measured the distance to the nearest FG-labeled neuron. This analysis allowed us to calculate the value of rA, the mean distance to the nearest neighbor between FG-labeled neurons in each mouse. We then calculated R, the ratio of rA to rE, where rE is the mean distance to the nearest neighbor expected from a randomly distributed population of FG-labeled neurons. The value of R can vary from 0 (for a distribution with maximum aggregation) to 2.1491 (for a perfectly uniform distribution). An R value of 1 corresponds to a random distribution of the cell population. The value of c, the standard variate for the normal curve, corresponds to the significance of a departure from the expected value of R = 1. The c values of 1.96 and 2.58 represent the 0.05 and 0.01 probability levels of statistical significance for measurements of a given population, respectively [54]. The R values of the dural afferent neurons from three mice were all close to 1, and their c values were all lower than 1.96 (Figure 6G), indicating that dural afferent neurons are randomly distributed in the V_1_/V_2_ divisions of the TG. This finding is consistent with a previous study showing a random distribution of TG neurons innervating the middle cerebral arteries in rats [25]. Secondly, we examined the spatial distribution of the TRPA1^+^ neurons in the V_1_/V_2_ divisions of the TG. As with the dural afferent neurons, the mean R value for the TRPA1^+^ neurons was also close to 1, suggesting that the TRPA1^+^ neurons are randomly distributed in the V_1_/V_2_ TG divisions (Figure 6G).

We proceeded to examine the spatial association between FG-labeled dural afferent neurons and TRPA1^+^ neurons using a modified nearest-neighbor measurement [55]. First, we tested whether TRPA1^+^ neurons are clustered around dural afferent neurons more than would be expected from a random distribution. We measured the distance (r) between each FG-labeled dural afferent neuron and its nearest TRPA1^+^ neuron and calculated the average nearest-neighbor distance (rA). We then computed the value of R, the ratio of rA to rE, where rE indicates the mean value of r for complete spatial independence between dural afferent neurons and TRPA1^+^ neurons. An R value of 1 indicates a lack of association (spatial independence) between the two cell populations, whereas an R value less than or greater than 1 suggests that the TRPA1^+^ TG neurons are more clustered than random (i.e., aggregation) or are more regularly distributed than random (i.e., avoidance), respectively, with respect to the FG-labeled neurons. The R values of three different mice were all greater than 1 (1.24 ± 0.15, Figure 6H). We tested the significance of this departure from spatial independence (i.e., R = 1) by calculating c, the standard variate of the normal curve. A mentioned above, c values of 1.96 or 2.58 represent the 0.05 and the 0.01 levels of significance for a two-tailed test, respectively [54,55]. The c values from three different mice were all greater than 2.4 (*p* < 0.05), suggesting that the TRPA1^+^ neuron population is distributed farther away from the dural afferent population than would be predicted by random association.

Since the number of FG-labeled neurons was approximately 2–3 fold greater than the number of TRPA1^+^ neurons in each TG section, we tested whether the TRPA1^+^ neurons cluster around a subgroup of dural afferent neurons but not the entire dural afferent population. Accordingly, we measured the distance between each TRPA1^+^ neuron and its nearest FG-labeled neuron and calculated the value of R as a measure of the spatial association. The R values obtained from three mice ranged from 0.46 to 0.74 (Figure 6H). The c values from these three mice were all greater than 7.5 (*p* < 0.0001), indicating a significant departure from spatial independence, leading to aggregation.

How close are the TRPA1^+^ neurons to their nearest FG-labeled dural afferent neuron? We found that the mean distance between the TRPA1^+^ neurons and the nearest FG-labeled neuron was 56 ± 2 arbitrary units (measured between the centers of the two cells). This distance was 1.7 ± 0.1 fold the mean distance between the TRPA1^+^ neurons and their closest neuron (32 ± 1 arbitrary units). Thus, there was approximately one neuron separating each TRPA1^+^ neuron from its nearest FG-labeled dural afferent neuron. On the other hand, we found that the mean distance between the closest pairs of TRPA1^+^ neurons or pairs of FG-labeled dural afferents was significantly greater than the distance between the TRPA1^+^ neurons and their closest FG-labeled neuron (101 ± 3 versus 77 ± 5 arbitrary units, respectively; *p* < 0.05, one-way ANOVA with post hoc Bonferroni test). Taken together, we conclude that the TRPA1^+^ neurons are clustered around some, but not all, of the dural afferent neurons in the TG. It is possible that the neurotransmitters and neuropeptides that are released from the soma of TRPA1^+^ neurons have a higher likelihood of cross-exciting this subpopulation of dural afferent neurons within the TG.

### TRPM8 channels are not expressed in dural afferent neurons

TRPM8 channel is a member of thermo-TRP family and transduces the cooling and cold sensations in mice [56-60]. Previous studies indicated that TRPM8-expressing neurons innervate both the skin and visceral organs [61-63]. Here, we used mice expressing farnesylated enhanced green fluorescent protein (EGFPf) at one TRPM8 locus (*TRPM8*^*EGFPf/+*^, [61]) to investigate whether TRPM8 is expressed in dural afferent neurons. All of the EGFPf-expressing (EGFPf^+^) dorsal root ganglion (DRG) neurons from *TRPM8*^*EGFPf/+*^ mice respond to both cold and menthol, indicating that the EGFP signal corresponds well with endogenous TRPM8 expression [61].

**Figure 7:**
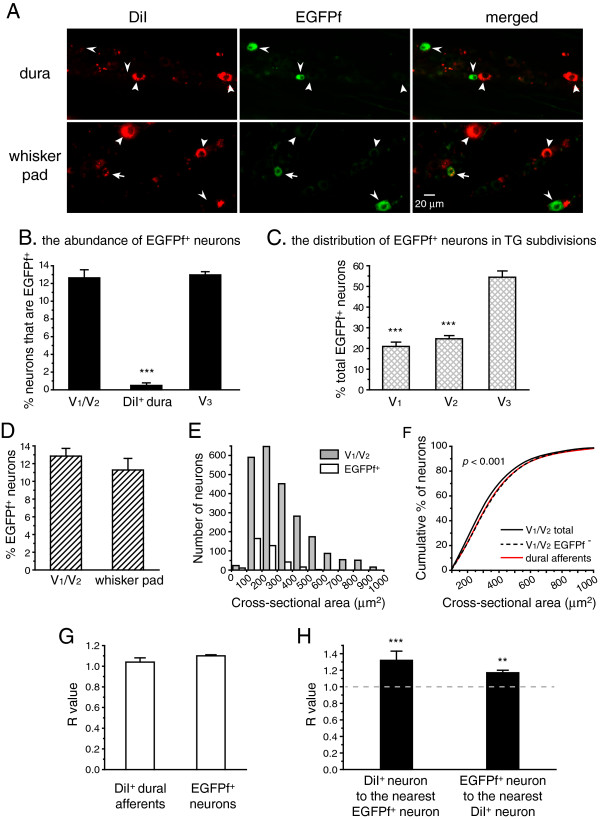
**The dural afferent neuron population lacks TRPM8-expressing neurons. **(**A**) Representative images of TG sections from *TRPM8*^*EGFPf/+*^ mice following dural DiI application or intradermal DiI injection at the whisker pad. The thick and thin arrowheads indicate DiI^+^ and EGFPf^+^ neurons, respectively. The arrows in the lower row indicate a DiI^+^ skin afferent neuron that is also EGFPf^+^. (**B**) The percentage of EGFPf^+^ neurons that are in the V_1_/V_2_ TG, the V_3_ TG and the DiI^+^ dural afferent neuron population (n = 3 mice, on average, 480 V_1_/V_2_ neurons, 472 V_3_, neurons and 206 DiI^+^ neurons were counted from each mouse; *** *p* < 0.001, two-tailed *t*-test, V_1_/V_2_ group versus DiI^+^ dura group). (**C**) The fraction of EGFPf^+^ neurons in the three TG divisions (n = 4 mice; on average, 1210 EGFPf^+^ neurons were counted from each mouse; *** *p* < 0.001, one-way ANOVA with post hoc Bonferroni test, all compared with the V_3_ distribution). (**D**) The percentage of EGFPf^+^ V_1_/V_2_ TG neurons and DiI^+^ neurons innervating the skin over the whisker pad (n = 4 mice, on average, 448 V_1_/V_2_ neurons and 76 DiI^+^ neurons were counted from each mouse). (**E**) The sizes of EGFPf^+^ neurons (n = 363 neurons pooled from five mice) are significantly smaller than those of the neurons in the V_1_/V_2_ divisions of the TG (n = 2403 neurons pooled from 5 mice, *p* < 0.001, Mann–Whitney U test). **(F)** Cumulative distributions of the cross-sectional areas of the total TG neuron populations in the V_1_/V_2_ divisions (the same neurons as in **E**), the EGFPf^−^ V_1_/V_2_ TG neurons (n = 2040 neurons pooled from five mice), and the FG^+^ dural afferent neurons (the same neurons as in Figure 1**F**). The sizes of EGFPf^−^ V_1_/V_2_ TG neurons are comparable to those of the dural afferent neurons, and both populations are significantly larger than the total V_1_/V_2_ neurons (*p* < 0.001, Kruskal-Wallis ANOVA with Dunn’s post hoc test). (**G**) Nearest-neighbor measurement shows that both DiI^+^ dural afferent neurons and EGFPf^+^ neurons are randomly distributed in the TG (n = 182 EGFPf^+^ neurons and 619 DiI^+^ dural afferent neurons from three mice, the same neurons as in **B**). (**H**) A modified nearest-neighbor measurement shows that the EGFPf^+^ neurons are located farther away from the dural afferent population than would be predicted by random association, and vice versa (the same neurons as in **G**).

We used DiI to retrogradely label the dural afferent neurons in *TRPM8*^*EGFPf/+*^ mice. Remarkably, we found an almost complete segregation of EGFPf fluorescence and the DiI signal in the TG (Figure 7A, B). In fact, of the 619 DiI-labeled dural afferent neurons measured from three *TRPM8*^*EGFPf/+*^ mice, only three neurons were EGFPf^+^. To determine whether this segregation is due to a lack of TRPM8-expressing neurons in the V_1_/V_2_ TG divisions, we examined both the distribution and abundance of EGFP^+^ neurons in all of the TG divisions of the *TRPM8*^*EGFPf/+*^ mice. Of all of the EGFPf^+^ neurons that we counted, approximately half were localized in the V_3_ division of the TG, and the remaining half were distributed uniformly between the V_1_ and V_2_ divisions (Figure 7C, *p* < 0.001 compared with the V_3_ division, one-way ANOVA with post hoc Bonferroni test). This result is in agreement with a previous report that TRPM8 expression is enriched in the V_3_ division of the TG [43]. We next measured the abundance of EGFPf^+^ neurons in the TG divisions. The percentages of TG neurons expressing EGFPf were similar between the V_1_/V_2_ and V_3_ divisions (12.6 ± 0.9% and 12.9 ± 0.4%, respectively, Figure 7B). Thus, it is unlikely that the lack of EGFPf^+^ neurons in the dural afferents arose from a low abundance of TRPM8-expressing neurons in the V_1_/V_2_ TG divisions. Notably, the overall abundance of EGFPf^+^ neurons in the TG was consistent with previous studies using anti-TRPM8 antibodies [56,64], further validating the EGFPf signal as a marker of endogenous TRPM8 expression. 

To test whether TRPM8-expressing neurons are deficient in either their ability to take up DiI at terminals and/or transporting DiI to the soma, we injected DiI into the skin over the whisker pad in *TRPM8*^*EGFPf/+*^ mice. The percentage of EGFPf^+^ neurons in the DiI-labeled skin afferents (11.3 ± 1.3%) was comparable to that of the V_1_/V_2_ neurons (12.8 ± 0.9%, Figure 7A, D), indicating that EGFPf^+^ neurons take up and transport DiI as efficiently as other TG neuron populations.

We showed above that the sizes of the dural afferent neurons are significantly larger than those of the V_1_/V_2_ TG neurons (Figure 1E, F). We wondered whether this difference stems from an absence of TRPM8-expressing neurons in the dural afferents. To address this question, we examined the size distribution of EGFPf^+^ neurons in the V_1_/V_2_ division of TG. The mean cross-sectional area of the EGFPf^+^ neurons in the V_1_/V_2_ was 223 ± 11 μm^2^ (n = 363 neurons pooled from five mice), which was significantly smaller than that of the total V_1_/V_2_ TG neuron population (339 ± 1 μm^2^, n = 2403 neurons pooled from five mice, *p* < 0.001, Mann–Whitney *U* test, Figure 7E). This result is consistent with previous reports that TRPM8 is expressed predominantly in small-diameter primary afferent neurons [45,59-62,64]. This finding led us to predict that the absence of TRPM8-expressing neurons in the dural afferent population could shift its size distribution towards the size of the EGFPf-negative (EGFPf^−^) V_1_/V_2_ TG neurons. Because the DiI-labeled neurons exhibited a punctate pattern of fluorescence, we found it difficult to accurately calculate their cross-sectional area. We therefore compared the sizes of the total and the EGFPf^−^ V_1_/V_2_ TG neurons with the sizes of the FG-labeled dural afferent neurons (the solid black line in Figure 1F). As expected, the sizes of the EGFPf^−^ V_1_/V_2_ TG neurons were similar to those of the FG-labeled dural afferent neurons (Figure 7F, *p* = 0.95, Kruskal-Wallis ANOVA with Dunn’s post hoc test); however, both neuron populations were significantly larger than the total V_1_/V_2_ neuron population (*p* < 0.001). Taken together, our results indicate that more than 10% of the neurons in the TG have a small-diameter soma and express TRPM8 but do not innervate the dura. This finding may account for the larger sizes of dural afferent neurons relative to the sizes of the total V_1_/V_2_ TG neuron population.

We proceeded to assess the spatial distribution of the DiI-labeled dural afferent neurons and the EGFPf^+^ neurons in the V_1_/V_2_ divisions of the TG using nearest-neighbor measurement [54]. The R values of the dural afferent neurons as well as the EGFPf^+^ neurons were all close to 1 (Figure 7G), and the c values were all less than 1.96, indicating that both the dural afferent neurons and the TRPM8-expressing neurons are distributed randomly in the V_1_/V_2_ divisions of the TG. We then investigated the spatial association between the DiI^+^ dural afferent neurons and the EGFPf^+^ neurons using the modified nearest-neighbor measurement [55]. We first tested whether the EGFPf^+^ neurons were randomly distributed, were more clustered than random (i.e., aggregation), or were more regularly distributed than random (i.e., avoidance) relative to the DiI-labeled dural afferent neurons. As shown in Figure 7H, the R values from three separate mice were all greater than 1 (1.32 ± 0.11), and the c values were all greater than 5.8 (*p* < 0.001), suggesting that the EGFPf^+^ neurons are located further from the dural afferent population than would be predicted by random association. Next, we tested whether the EGFPf^+^ neurons are more clustered around a subpopulation of dural afferent neurons (as is the case for the TRPA1^+^ neurons). Accordingly, we measured the distance between each EGFPf^+^ neuron and its nearest DiI-labeled neuron and then calculated the R value. The R values obtained from three mice were all greater than 1 (1.17 ± 0.02, Figure 7H), and the c values were all greater than 3.1 (*p* < 0.01). In fact, the mean distance between the EGFPf^+^ neurons and their nearest DiI-labeled neuron was 163 ± 11 arbitrary units, which is 3.9 ± 0.3 fold greater than the mean distance between the EGFPf^+^ neurons and their adjacent neuron (33 ± 2 arbitrary units). Taken together, we conclude that TRPM8-expressing neurons and dural afferent neurons are located farther away from each other than would be predicted by random association in the V_1_/V_2_ divisions of the TG. It is therefore unlikely that these two populations of neurons cross-excite each other within the TG.

## Discussion

In this study, we used two fluorescent tracers, FG and DiI, to retrogradely label dural afferent neurons in adult mice. This approach allowed us to quantitatively compare both the size distribution and the protein expression profiles of dural afferent neurons with those of the total TG neuron population and the facial skin afferents. Our results show that the TG neurons that innervate the dura over the SSS are predominantly localized in the V_1_/V_2_ divisions of the TG. The sizes of dural afferent neurons in mice are significantly larger than those of the V_1_/V_2_ TG neurons and the facial skin afferents, which is consistent with previous studies using rats [25,28,29].

A substantial percentage of dural afferent neurons bind IB4, suggesting that these neurons belong to the non-peptidergic PAN population. These neurons likely express P2X_3_ receptors and mediate the pronociceptive effects of ATP [65]. In contrast, the percentage of CGRP^+^ dural afferent neurons (~15%) was only half those of the V_1_/V_2_ TG neurons or the facial skin afferents. We excluded the possibility that this result is due to a low efficiency of CGRP^+^ neurons to take up FG and DiI at their terminals and/or transport the tracers to the soma. Our result is in contrast with a previous study quantifying the abundance of CGRP^+^ neurons in TG neurons that innervate the intracranial arteries in rats [37]. O’Connor and van der Kooy (1988) reported that 32% of the TG neurons that project to the cerebral vasculature express CGRP, which is much higher than the 23% of CGRP^+^ neurons that was observed in the entire TG. Differences in the animal species (rat versus mouse) and target tissues (cerebral vasculature versus dura) between the two studies may account for this discrepancy. We suspect that one crucial difference may be the way in which the tissue was prepared prior to immunostaining with the anti-CGRP antibody. In the previous study, the TG tissues were organ-cultured in serum-free medium containing colchicine for 9–12 hours before fixation and immunostaining. The colchicine pretreatment and/or the organ culture procedure *per se* may have preferentially increased the CGRP levels in the TG neurons that innervate cerebral arteries. Indeed, recent studies have shown enhanced CGRP expression in rat TG neurons during cell and organ cultures in serum-free medium [66,67]. On the other hand, in our approach, we may have underestimated the number of CGRP^+^ neurons in the dural afferents. It is possible that dural afferent neurons exhibit enhanced trafficking of CGRP-containing vesicles towards the terminals and/or have enhanced exocytosis of CGRP, either of which could result in a depletion of somatic CGRP stores. The dense innervation of CGRP^+^ fibers at the meninges and cerebral arteries in rodents is well-documented [29,34-36]. On the other hand, acutely-dissociated dural afferent neurons do not exhibit spontaneous action potential firing [27]. In fact, injection of a depolarizing current elicits a significantly higher number of action potentials in skin afferent neurons than in dural afferents, suggesting the lower excitability of the latter population [68]. This argues against enhanced exocytosis in dural afferent neurons. Further work is needed to resolve the discrepancy between our data and previous studies.

**Figure 8:**
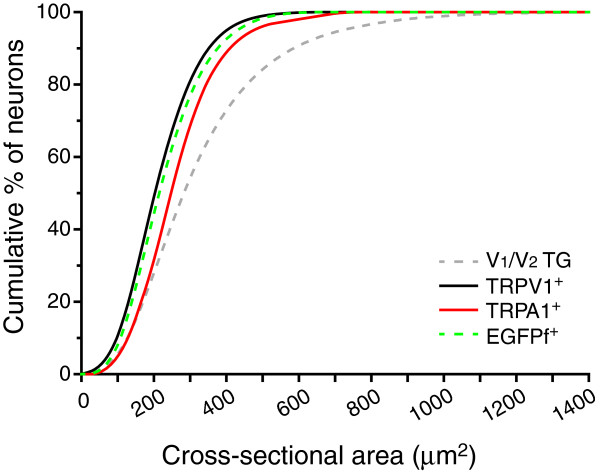
**Overlay of the cumulative distributions of the TRPV1**^**+**^**, TRPA1**^**+**^**and TRPM8-expressing neurons in the V**_**1**_**/V**_**2**_**divisions of the TG.** Cumulative distribution of the cross-sectional areas of the total TG neurons in the V_1_/V_2_ divisions (dashed gray line, the same neurons as in Figure 1**F**), the TRPV1^+^ neurons (solid black line, the same neurons as in Figure 5**E**), the TRPA1^+^ neurons (red line, the same neurons as in Figure 6**E**), and the TRPM8-expressing neurons (dashed green line, the same neurons as in Figure 7**E**). A Kruskal-Wallis ANOVA with Dunn’s post hoc test reveals that the sizes of TRPV1^+^ neurons are the smallest of the three populations of TG neurons (*p* < 0.001 and *p* < 0.05 compared with the TRPA1^+^ and EGFPf^+^ groups, respectively). In contrast, the sizes of TRPA1^+^ neurons are significantly larger than those of the TRPV1^+^ and TRPM8-expressing neurons (*p* < 0.001 and *p* < 0.05, respectively).

The primary purpose of this study was to quantitatively evaluate the expression of TRPV1, TRPA1, and TRPM8 channels in dural afferent neurons. First, we found that the percentages of TG neurons that express these channels are consistent with previous studies [7,43,45,51,52,56,58-60,64]. The size distributions of TRPV1^+^, TRPA1^+^, and TRPM8-expressing TG neurons were also similar to previous reports. As summarized in Figure 8, the sizes of TRPV1^+^ neurons are the smallest among the three populations of TG neurons (*p* < 0.001 and *p* < 0.05 compared with the TRPA1^+^ and EGFPf^+^ groups, respectively). The cross-sectional areas of the TRPA1^+^ neurons are significantly larger than those of the TRPV1^+^ and TRPM8-expressing neurons (*p* < 0.001 and *p* < 0.05, respectively, Kruskal-Wallis ANOVA with Dunn’s post hoc test) neurons. This finding is consistent with previous reports that TRPA1 is expressed in the subpopulation of TRPV1 neurons that have a relatively large diameter soma [45,69]. Secondly, the percentage of TRPV1^+^ dural afferent neurons in our study is similar to that in a previous report [6] but is ~20% lower than that in the total V_1_/V_2_ TG neuron population. On the other hand, the percentage of TRPA1^+^ neurons in the dural afferents is comparable to that in the V_1_/V_2_ TG population. Taken together, our results suggest that compared with total TG neurons, dural afferent neurons contain a smaller fraction of TRPV1^+^ cells that do not express TRPA1 channels. 

A noteworthy finding in this study is the absence of TRPM8-expressing neurons in the dural afferent population, despite the fact that TRPM8 is expressed in more than 10% of both the total and skin afferent TG neurons. This may, at least partially, account for the difference in size distribution between the dural afferents and the total TG neurons. Furthermore, results from our nearest-neighbor measurement predicte that TRPM8-expressing neurons and dural afferent neurons are farther away from each other than would be predicted by random association, suggesting that TRPM8 channels may have only a small, or no, contribution to the activation of PANs in the headache circuit. On the contrary, not only are TRPA1 channels expressed in a small population of dural afferent neurons, TRPA1^+^ neurons are also clustered around some, but not all, dural afferent neurons in the TG. Previous studies have shown that stimulating PANs elicits the somatic release of ATP, substance P, and CGRP [18-22]. These somatically released neurotransmitters and neuropeptides may account for the cross-depolarization and cross-excitation between PANs that have been observed in rat DRG and nodose ganglia [14-17]. Our data suggest that TRPA1 channels may directly excite dural afferent neurons as well as play a role in the intraganglionic cross-excitation of dural afferents. These results are in agreement with recent studies showing that the intranasal administration of TRPA1 agonists stimulates CGRP release and increases meningeal blood flow [12,13]. Our results provide anatomical evidence to support a possible scenario by which TRPA1^+^ neurons innervating the nasal mucosa may cross-excite nearby dural afferent neurons. Future experiments are necessary to directly test this possibility.

That said, it is possible that our method may not have been sufficiently sensitive to detect low expression levels of TRPM8 and/or TRPA1 channels in the TG and dural afferent neurons. Indeed, an *in situ* hybridization study showed a low level of TRPM8 expression in medium-sized DRG neurons in rats [43]; in our study, all of the EGFPf^+^ neurons belonged to the small-sized TG population (< 600 μm^2^ cross-sectional area; Figure 7E). The abundance of TRPA1 channels in our study (in 6 ~ 7% of the TG neurons; Figure 6B, C) is consistent with some of previous results [45,51] but is much lower than the 20 ~ 25% that has been reported in other studies [44,46,47]. Kwan et al. measured *Trpa1* mRNA and found that mouse DRG neurons of all sizes express TRPA1 [53]. Moreover, a recent functional study found that 40% of rat dural afferent neurons respond to TRPA1 agonists [11]. Thus, it is possible that the TRPA1^+^ population in our study preferentially contains neurons that express high levels of TRPA1 channels. Taken together, whether and how various TRP channels contribute to the activation and/or sensitization of PANs in the headache circuit merits further investigation. Our study has established an anatomical foundation upon which mouse models can be used to address the role of TRP channels in headache pathophysiology.

## Conclusions

In this study, we have quantitatively measured the size distributions and protein expression profiles of dural afferent neurons in adult mice. We provide evidence that a substantial fraction of dural afferent neurons bind IB4, whereas the percentage of CGRP^+^ dural afferent neurons is significantly lower than in total TG neuron population. Both TRPV1 and TRPA1 channels are expressed in dural afferent neurons. In addition, TRPA1^+^ neurons are clustered around a subset of dural afferent neurons, suggesting that they may have a higher probability of generating cross-excitation within the TG. Interestingly, TRPM8-expressing neurons are virtually absent in the dural afferent population, nor do they cluster around dural afferent neurons. We postulate that this lack of TRPM8-expressing neurons may partially account for the larger sizes of dural afferent neurons relative to those of the total TG neuron population.

## Methods

### Experimental animals

Eight-to-twelve-week old mice on a C57BL/6 background were used in this study. The care and use of mice were in accordance with the guidelines of the Animal Study Committee at Washington University in St. Louis. Hemizygous mice expressing EGFPf at the TRPM8 locus were obtained by crossing heterozygous breeders. Mice were genotyped by PCR of their tail DNA as described previously [61].

### Retrograde labeling of TG neurons innervating the dura or the facial skin

Mice were anesthetized with 3-4% isoflurane in an induction chamber until the loss of the righting reflex. The mice were then mounted on a Stoelting stereotaxic apparatus and placed on a 37 °C circulating water warming pad to maintain core body temperature. Anesthesia was maintained by 1.5-2% isoflurane through a nose cone. A longitudinal skin incision was made to expose the cranium, and a craniectomy (~2 mm in diameter) was made using a cooled dental drill in the skull overlying the SSS, leaving the underlying dura exposed but intact. Topical lidocaine was applied to the skin and skull to prevent the activation and/or sensitization of the primary afferent neurons. To prevent spreading of the tracer to other peripheral sites, a sterile polypropylene ring was sealed to the skull surrounding the exposed dura using a mixture of dental cement powder (Stoelting 51459) and superglue adhesive [70]. The viscosity of the dental cement/superglue mixture prevented spreading to the exposed dura. After waiting 5–10 min for the mixture to solidify, we applied 7 μl of DiI solution (20 mg/ml in PBS with 10% DMSO, Invitrogen) or FG (2% in 0.9% saline, Fluorochrome) onto the exposed dura. Subsequently, the dura was covered with a sterile polypropylene cap that was secured over the ring using the dental cement/superglue mix. The skin incision was closed using stainless steel wound clips. After recovery from anesthesia, the mice were housed individually in the animal facility for five (for FG labeling) or ten days (for DiI labeling) to allow the transport of the tracer to the somata in the TG.

To label the TG neurons innervating the facial skin, we shaved the skin at the periorbital region (between the two eyes) and injected 7 μl of DiI or FG solution intradermally. The needle was held nearly parallel to the skin and inserted ~1 mm into the skin. The injection was performed slowly over a period of ~1 minute. In some *TRPM8*^*EGFPf/+*^ mice, we injected 7 μl DiI intradermally into the skin over the whisker pad. After the injection, the mice were housed in the animal facility for five (for FG labeling) or ten days (for DiI labeling) to allow for transport of the tracer to the somata in the TG.

To label both the dural and skin afferent neurons in the same mouse, we first applied 7 μl of DiI onto the dura and then injected 7 μl FG solution intradermally into the periorbital skin five days after the craniectomy. The mice were housed individually in the animal facility for an additional five days before being euthanized.

### Tissue preparation and immunohistochemistry (IHC)

The mice were euthanized by barbiturate overdose (200 mg/kg, i.p.) and were transcardially perfused with 0.1 M phosphate-buffered saline (PBS) followed by 4% formaldehyde in 0.1 M phosphate buffer, pH 7.4 (PB) for fixation. The TG tissues were removed, post-fixed for two hours, and then protected overnight in 30% sucrose in 0.1 M PB. The ganglia were sectioned at 20 μm using a cryostat, mounted on Superfrost Plus glass slides and stored at −20 °C. One in every three sections (cut approximately every 60 μm) was processed for each IHC experiment.

For IHC of the FG-labeled TG, the sections were dried at room temperature (RT), washed three times in 0.01 M PBS and incubated in blocking buffer (0.01 M PBS with 10% normal goat serum (NGS) and 0.3% Triton X-100) for 1 hr at RT. The sections were then incubated overnight with primary antibodies that were diluted in blocking buffer at 4 °C. Following 3–5 10-min washes in 0.01 M PBS containing 1% NGS and 0.3% triton and blocking for 1 hr, the sections were incubated with the secondary antibodies in blocking buffer at RT for 1 hour, and then washed three times in 0.01 M PBS. The sections were cover-slipped using Fluoromount-G Slide Mounting Medium (Electron Microscopy), sealed with nail polish, and stored at 4 °C. For IHC of the DiI-labeled TG, the concentration of Triton X-100 in all solutions was reduced to 0.03% to preserve the DiI signal [38].

The primary antibodies against CGRP (Millipore) and TRPV1 (Neuromics) were used at 1:1000 dilution. Two antibodies against distinct extracellular domains of TRPA1 were combined and used at 1:50 dilution as described previously [51]. The Alexa Fluor 568- and 488-conjugated goat anti-rabbit secondary antibodies (Invitrogen) were used at 1:1000 dilution. To measure IB4 affinity, the sections were incubated with 2 μg/ml Alexa Fluor 594-conjugated IB4 in blocking buffer at 4 °C overnight.

### Image acquisition and data analysis

Images of the entire TG section were captured using an Olympus NanoZoomer Whole-Slide Imaging System at the Alafi neuroimaging core facility at Washington University Medical School. High-power images of the TG sections were examined and captured through a 20x objective on a Nikon TE2000S inverted epifluorescence microscope equipped with a CoolSnapHQ^2^ camera (Photometrics). Cross-sectional somatic area was measured using SimplePCI software (Hamamatsu). Figures were prepared using Origin 8.1 (OriginLab). The individual images were adjusted for contrast and brightness. No other manipulations were made to the images.

### Statistical analysis

All summary data are reported as the mean ± standard error of the mean (SEM). Statistical tests were performed using Statistica10 software (StatSoft). Differences with *p <* 0.05 were considered to be statistically significant. A two-tailed Student’s *t*-test or one-way analysis of variance (ANOVA) with post hoc Bonferroni test was used as the parametric statistical test where appropriate. The non-parametric Mann–Whitney *U* test or the Kruskal-Wallis ANOVA with Dunn’s post hoc test was used where appropriate to analyze the differences in the soma size distribution.

### Nearest-neighbor measurement

The nearest-neighbor measurement was used to determine whether cells in a given TG population (e.g., dural afferent neurons) were distributed randomly or were clustered together [54]. Briefly, the distance between two cells (r) was measured from the center of the cell in question to the center of its corresponding nearest neighbor, and the mean r value was computed using the following equation: $\text{rA}=\frac{\text{Σ}\text{r}}{\text{N}}$ , with N being the total number of cells in question. The mean r value for a random distribution of cells was calculated using the following equation: $\text{r}\text{E}=\frac{1}{2\sqrt{\text{ρ}}}$, with ρ being the density of the cell population of interest. The ratio $\text{R}=\frac{\text{rA}}{\text{rE}}$ of the observed mean distance to the expected mean distance provides a measure of the degree to which the distribution pattern of the observed population deviates from random expectation. R can range in value from 0 (for a distribution with maximum aggregation) to 2.1491 (for a perfectly uniform distribution). An R value of 1 corresponds to a random distribution of the cell population. The significance of the departure from random expectation was tested by the standard variate of the normal curve using the following equation: $\text{c}=\frac{\text{rE}-\text{rA}}{\sigma \text{rE}}$, where σrE is the standard error of rE. The c values 1.96 and 2.58 represent the 0.05 and the 0.01 levels of significance, respectively, for a two-tailed test.

We used a modified nearest-neighbor measurement to assess the spatial association between two discrete populations of TG neurons (for example, between dural afferent neurons and TRPM8-expressing neurons) [55]. Briefly, the value of rA was obtained by averaging the distances between a given cell in one population and its nearest neighbor in the other population (i.e., the nearest-neighbor distance). The mean r value for complete spatial independence between cells in the two populations was calculated using the following equation: $\text{r}\text{E}=\frac{\text{n}1}{2\sqrt{\text{ρ}2}}+\frac{\text{n}2}{2\sqrt{\text{ρ}1}}$ , with n1 and n2 being the relative proportions of the two cell populations (n1 + n2 =1), and ρ1 and ρ2 being the densities of the two respective cell populations. The ratio $\text{R}=\frac{\text{rA}}{\text{rE}}$ provides a measure of the spatial association between two populations of cells, with R = 1 indicating a lack of association (i.e., spatial independence). An R value less than or greater than 1 corresponds to a spatial association between two cell populations that is more clustered than random (i.e., aggregation) or more regular than random (i.e., avoidance), respectively. The significance of the departure from the expected spatial independence was tested by the standard variate of the normal curve as follows: $\text{c}=\frac{\text{rE}-\text{rA}}{\sigma \text{rE}}$, where σrE is the standard error of rE. The c values 1.96 and 2.58 represent the 0.05 and the 0.01 levels of significance, respectively, for a two-tailed test.

## Abbreviations

ANOVA: Analysis of variance; CGRP: Calcitonin gene-related peptide; CGRP-ir: CGRP immunoreactivity; CGRP^+^: CGRP-expressing; DiI: 1,1'-dioctadecyl-3,3,3',3'-tetramethylindocarbocyanine perchlorate; DiI^+^: DiI-labeled; DRG: Dorsal root ganglion; EGFPf: Farnesylated enhanced green fluorescent protein; EGFPf^+^: EGFPf-expressing; EGFPf^−^: EGFPf-negative; FG: Fluorogold; FG^+^: FG-labeled; IB4: Isolectin B4; IB4^+^: IB4-labeled; IHC: Immunohistochemistry; NGS: Normal goat serum; PB: Phosphate buffer; PBS: Phosphate-buffered saline; PAN: Primary afferent neuron; RT: Room temperature; SEM: Standard error of the mean; SSS: Superior sagittal sinus; TG: Trigeminal ganglion; TRP channel: Transient receptor potential channel; TRPA1: Transient receptor potential cation channel subfamily A member 1; TRPA1-ir: TRPA1-immunoreactivity; TRPA1^+^: TRPA1-expressing; TRPM8: Transient receptor potential channel melastatin 8; TRPM8^EGFPf/+^: Hemizygous mice expressing EGFPf protein at the TRPM8 locus; TRPM8-ir: TRPM8-immunoreactivity; TRPV1: Transient receptor potential cation channel subfamily V member 1; TRPV1-ir: TRPV1 immunoreactivity; TRPV1^+^: TRPV1-expressing; V_1_: The ophthalmic division of the TG; V_2_: The maxillary division of the TG; V_3_: The mandibular division of the TG.

## Competing interests

The authors declare no competing interests.

## Authors’ contributions

DH and YQC designed the research. DH performed the experiments. AD and GMS contributed new reagents. DH, SL, and YQC contributed to the data acquisition, analysis, and interpretation. DH, SL, GMS, and YQC wrote the manuscript. All authors read and approved the final manuscript.

## Author details

^1^Washington University Pain Center and Department of Anesthesiology, Washington University School of Medicine, St. Louis, MO 63110, USA. ^2^Department of Biological Structure, University of Washington, Seattle, WA 98195, USA.
